# The association between carotid blood flow and resting-state brain activity in patients with cerebrovascular diseases

**DOI:** 10.1038/s41598-021-94717-0

**Published:** 2021-07-27

**Authors:** Takahiro Matsumoto, Hideyuki Hoshi, Yoko Hirata, Sayuri Ichikawa, Keisuke Fukasawa, Tomoyuki Gonda, Jesús Poza, Víctor Rodríguez-González, Carlos Gómez, Yoshihito Shigihara

**Affiliations:** 1 Department of Neurosurgery, Kumagaya General Hospital, Kumagaya, 360-8567 Japan; 2 Precision Medicine Centre, Hokuto Hospital, Kisen-7-5 Inadacho, Obihiro, Hokkaido 080-0833 Japan; 3 Clinical Laboratory, Kumagaya General Hospital, Kumagaya, 360-8567 Japan; 4 Department of Rehabilitation, Kumagaya General Hospital, Kumagaya, 360-8567 Japan; 5 Biomedical Engineering Group, Higher Technical School of Telecommunications Engineering, University of Valladolid, Castilla y León, 47011 Valladolid, Spain; 6 Centro de Investigación Biomédica en Red en Bioingeniería, (CIBER-BBN), Biomateriales y Nanomedicina, Castilla y León, 47011 Valladolid, Spain; 7 Instituto de Investigación en Matemáticas (IMUVA), University of Valladolid, Castilla y León, 47011 Valladolid, Spain; 8 Precision Medicine Centre, Kumagaya General Hospital, Kumagaya, 360-8567 Japan

**Keywords:** Neuroscience, Cognitive ageing, Cognitive neuroscience, Neural ageing, Ageing, Cardiovascular biology, Circulation, Neurological disorders, Cerebrovascular disorders, Dementia, Neurovascular disorders, Stroke

## Abstract

Cerebral hypoperfusion impairs brain activity and leads to cognitive impairment. Left and right common carotid arteries (CCA) are the major source of cerebral blood supply. It remains unclear whether blood flow in both CCA contributes equally to brain activity. Here, CCA blood flow was evaluated using ultrasonography in 23 patients with cerebrovascular diseases. Resting-state brain activity and cognitive status were also assessed using magnetoencephalography and a cognitive subscale of the Functional Independence Measure, respectively, to explore the relationships between blood flow, functional brain activity, and cognitive status. Our findings indicated that there was an association between blood flow and resting-state brain activity, and between resting-state brain activity and cognitive status. However, blood flow was not significantly associated with cognitive status directly. Furthermore, blood velocity in the right CCA correlated with resting-state brain activity, but not with the resistance index. In contrast, the resistance index in the left CCA correlated with resting-state brain activity, but not with blood velocity. Our findings suggest that hypoperfusion is important in the right CCA, whereas cerebral microcirculation is important in the left CCA for brain activity. Hence, this asymmetry should be considered when designing appropriate therapeutic strategies.

## Introduction

Dementia is a syndrome characterised by progressive cognitive impairment due to diverse brain diseases. Alzheimer's disease is the most prevalent cause of dementia followed by cerebrovascular diseases, which are typically termed ‘vascular dementia’^1,2^. Cerebrovascular diseases lead to cerebral hypoperfusion and stroke^3^. Hypoperfusion itself changes brain activity^4^ and leads to cognitive impairment^5–7^. Cerebral blood supply depends on two pairs of arteries, namely, the left and right internal carotid arteries and the vertebral arteries, with three-quarters of the blood supplied by the internal carotid artery pair^8^. The internal carotid artery is a branch of the common carotid artery (CCA) and its blood flow velocity has been associated with cognition in older adults^9,10^. Both sides of the CCA supply blood mainly to the ipsilateral side of the cerebral hemisphere, with each hemisphere contributing differently to cognitive status^11–13^. In this context, we hypothesised that there was an asymmetrical association between the two sides (i.e. left and right) of the CCA in terms of brain activity and cognitive status.

Blood flow in the CCA can be measured using carotid ultrasonography, which is a non-invasive measurement method for hypoperfusion due to atherosclerosis. It provides information on blood flow velocities and other haemodynamic factors, such as downstream resistance. Resting-state brain activity can be measured using magnetoencephalography (MEG). Resting-state MEG measures spontaneous neural oscillations and is sensitive to cerebral hypoperfusion^14^, which reduces the amplitude and lowers the frequency of oscillatory activities^4,14^. Changes in resting-state brain activity are also associated with cognitive impairment^15–17^, which, in turn, is related to three major characteristic alterations: (1) enhanced low frequency oscillatory activity accompanied with attenuated high frequency oscillatory activity; (2) slowing down of the alpha peak frequency (so-called ‘shift-to-the-left of the alpha peak’); and (3) loss of irregularity of brain activity^15,17–19^. Cognitive status is generally assessed using neuropsychological tests, such as the Functional Independence Measure (FIM)^20–22^ and the Mini-Mental State Examination (MMSE)^10^. The FIM is used to evaluate the cognitive and motor status of patients with cerebrovascular diseases, especially during rehabilitation periods, whereas the MMSE is used for screening dementia in general. A previous study showed that cognitive impairments, due to cerebral hypoperfusion, affected the MMSE score^10^.

In this study, we aimed to determine whether there was an asymmetrical association between carotid artery blood flow, brain activity, and cognitive status. We investigated the association between carotid blood flow and both resting-state brain activity and cognitive status in patients with cerebrovascular diseases using carotid ultrasonography, MEG, and the FIM scale.

## Results

### Statistical analysis

In our analysis, we used two different statistical approaches, namely, bootstrapping correlation and linear mixed-effect model (LMEM) analysis. Bootstrapping correlation provides information concerning non-directional relationships between two parameters that are not relevant to the causality of the two parameters (i.e*.* a cause-effect relationship was not considered). In this case, we used the terms 'correlate/correlation'. LMEM analysis provides information concerning directional relationships between parameters (i.e*.* a cause-effect relationship was considered). In this case, we used the term 'influence'. We used the terms 'relationship/association' when both were mentioned.

### Within-modality association: ultrasonography, MEG, and FIM

**Figure 1:**
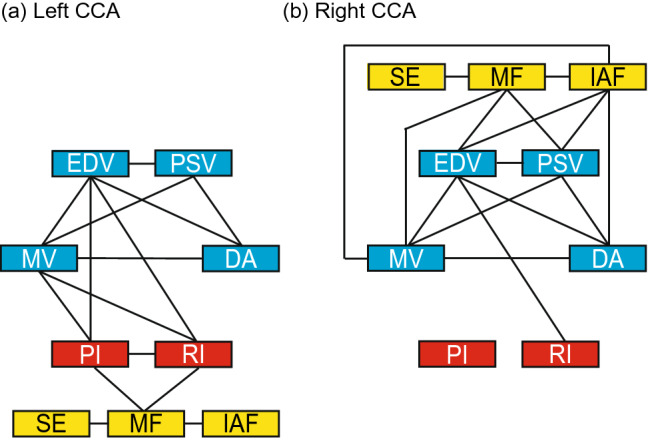
Schematic descriptions of relationships within/between ultrasonographic parameters and MEG spectral parameters. Lines represent a significant correlation. Blue, red, and yellow boxes represent local factors, downstream factors, and MEG spectral parameters, respectively. Biases in terms of patient age and sex were not considered in the relationships depicted here. CCA common carotid artery, D A diameter of artery, EDV end-diastolic velocity, IAF individual alpha frequency, MEG magnetoencepharography, MF median frequency, MV mean velocity, PI pulsatility index, PSV peak systolic flow velocity, RI resistance index, SE Shannon entropy.

To interpret the following results accurately, relationships between parameters within specific modalities were assessed. Concerning the carotid ultrasonography data, six parameters used in daily clinical practice were employed for analysis: diameter of artery (DA), peak systolic flow velocity (PSV), end-diastolic velocity (EDV), mean velocity (MV), the pulsatility index (PI), and the resistance index (RI). Two categories were formed, namely: DA, PSV, EDV, and MV, which were considered local factors of blood vessels; and PI and RI, which were considered downstream factors^23^. The four local factors significantly correlated with each other in both sides of the CCA (Table 1; Fig. 1), whereas the two downstream factors correlated in the left side of the CCA only. Several cross-category correlations were also found: EDV/MV and PI/RI correlated in the left side, and EDV and RI also correlated in the right side.

**Table 1:** Correlation matrix within sided ultrasonographic parameters.

	DA			PSV			EDV			MV			PI		
	*r*	P (unc.)	P (FDR)	*r*	P (unc.)	P (FDR)	*r*	P (unc.)	P (FDR)	*r*	P (unc.)	P (FDR)	*r*	P (unc.)	P (FDR)
**Left CCA**															
PSV	− 0.644	< 0.001*	< 0.001*												
EDV	− 0.339	0.035*	0.047*	0.646	< 0.001*	< 0.001*									
MV	− 0.548	0.001*	0.002*	0.793	< 0.001*	< 0.001*	0.907	< 0.001*	< 0.001*						
PI	− 0.054	0.388	0.416	0.083	0.346	0.400	− 0.632	< 0.001*	< 0.001*	− 0.492	0.003*	0.005*			
RI	− 0.132	0.271	0.339	0.027	0.446	0.446	− 0.712	< 0.001*	< 0.001*	− 0.475	0.007*	0.011*	0.927	< 0.001*	< 0.001*
**Right CCA**															
PSV	− 0.634	< 0.001*	< 0.001*												
EDV	− 0.660	< 0.001*	0.001*	0.809	< 0.001*	< 0.001*									
MV	− 0.722	< 0.001*	< 0.001*	0.923	< 0.001*	< 0.001*	0.944	< 0.001*	< 0.001*						
PI	0.252	0.104	0.196	0.152	0.235	0.320	− 0.175	0.161	0.243	− 0.107	0.256	0.320			
RI	− 0.055	0.335	0.377	0.110	0.162	0.243	− 0.310	0.002*	0.003*	− 0.083	0.394	0.394	− 0.018	0.352	0.377

*DA* diameter of artery, *CCA* common carotid arteries, *EDV* end-diastolic velocity, *MV* mean velocity, *P (unc.)* uncorrected *p* values of bootstrapping statistics, *PI* pulsatility index, *r* average correlation coefficient across bootstrap iterations, *PSV* peak systolic flow velocity, *RI* resistance index, *P (FDR)* *p* values of bootstrapping statistics with FDR-correction.

*A statistically significant correlation after FDR correction.

Concerning MEG, three spectral parameters were calculated from MEG signals to represent the properties of spontaneous neural oscillations, namely, median frequency (MF), individual alpha frequency (IAF), and Shannon entropy (SE)^17,24,25^. MF positively correlated with IAF (*r* = 0.856, P (false discovery rate [FDR]-corrected) < 0.001) and SE (*r* = 0.738, P (FDR) = 0.002). IAF did not significantly correlate with SE (*r* = 0.469, P (FDR) = 0.076).

FIM is an assessment tool for cognitive and motor status during rehabilitation for stroke^22^. It consists of two domains: a cognitive subscale (cognitive-FIM) and a motor subscale (motor-FIM). The correlation between cognitive-FIM and motor-FIM was not significant (*r* = 0.369, P (FDR) = 0.065).

### Between modality association: ultrasonography, MEG, and FIM

To investigate the asymmetrical effects of CCA on resting-state brain activity and cognitive status, ultrasonographic parameters of both sides of the CCA were compared with MEG spectral parameters and FIM scores separately.

**Figure 2:**
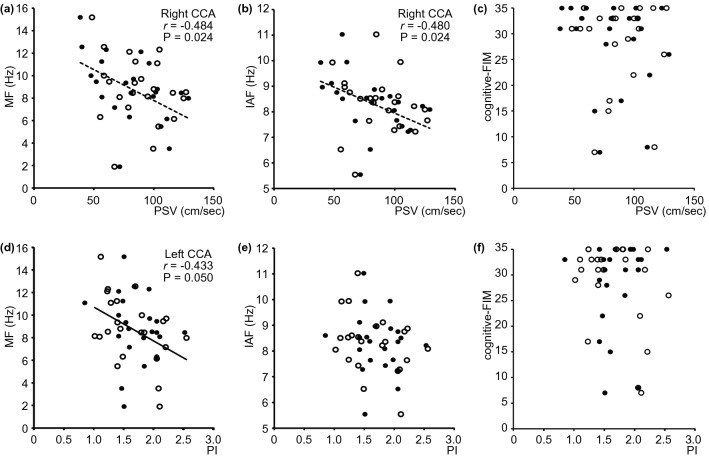
Scatterplots visualising the relationship between the ultrasonographic parameters [(**a**–**c**), PSV; and (**d**–**f**), PI] in either side of the CCA and the MEG spectral parameters and cognitive-FIM score [(**a**,**d**), MF; (**b**,**e**), IAF; and (**c**,**f**), cognitive-FIM]. Regression lines are added where there is a significant correlation between the parameters (bootstrapping statistics after FDR correction). Open dot, left CCA; filled dot, right CCA; solid regression line, left CCA; broken regression line, right CCA. Biases in terms of patient age and sex were not considered. *CCA* common carotid arteries, *FDR* false discovery rate, *FIM* functional independent measure, *IAF* individual alpha frequency, *MEG* magnetoencephalography, *MF* median frequency, *PI* pulsatility index, *PSV* peak systolic flow velocity.

In terms of the left CCA, ultrasonography downstream factors (PI and RI) showed stronger relationships with the MEG spectral parameter (MF) than with local factors (DA, PSV, EDV, and MV; Table 2; Figs. 1a, 2d,e). The PI and the RI in the left CCA negatively correlated with MF. Although the correlations did not reach the statistical threshold after FDR correction, PI/RI and IAF the RI and SE, and PI/RI and cognitive-FIM also showed mild correlations (P (uncorrected) < 0.05). When the biases from patients’ profiles (i.e., age and sex) had been eliminated, the RI continued to mildly influence MF (P (uncorrected) = 0.015; Table 3). No local factor (DA, PSV, EDV, and MV) in the left CCA correlated with or influenced any MEG spectral parameter after considering potential profile biases. Similarly, the associations between PI/RI and cognitive-FIM were not significant after controlling for profile biases (Table 3). After controlling for profile biases, no significant correlations or influences were found between local factors (DA, PSV, EDV, and MV) and cognitive-FIM and between ultrasonographic parameters in the left CCA and motor-FIM (Table 3).

**Table 2:** Correlation matrix between sided ultrasonographic parameters, MEG spectral parameters, and FIM.

	DA			PSV			EDV			MV		
	*r*	P (unc.)	P (FDR)	*r*	P (unc.)	P (FDR)	*r*	P (unc.)	P (FDR)	*r*	P (unc.)	P (FDR)
**Left CCA**												
MF	0.262	0.154	0.284	− 0.231	0.158	0.284	0.169	0.187	0.305	− 0.014	0.467	0.480
IAF	− 0.003	0.480	0.480	− 0.143	0.273	0.351	0.125	0.260	0.351	0.028	0.432	0.480
SE	0.293	0.107	0.241	0.063	0.368	0.442	0.288	0.045*	0.135	0.126	0.254	0.351
Mot	− 0.130	0.255	0.352	− 0.161	0.215	0.352	− 0.109	0.313	0.352	− 0.114	0.303	0.352
Cog	− 0.070	0.323	0.352	− 0.025	0.461	0.461	0.217	0.130	0.352	0.179	0.162	0.352
**Right CCA**												
MF	0.419	0.042*	0.095	− 0.484	0.007*	0.024*	− 0.421	0.013*	0.039*	− 0.506	0.004*	0.024*
IAF	0.358	0.018*	0.046*	− 0.480	0.005*	0.024*	− 0.390	0.006*	0.024*	− 0.475	0.002*	0.024*
SE	0.332	0.055	0.110	− 0.094	0.325	0.344	− 0.205	0.178	0.233	− 0.248	0.114	0.186
Mot	0.101	0.299	0.359	− 0.125	0.251	0.359	− 0.110	0.281	0.359	− 0.142	0.230	0.359
Cog	0.177	0.085	0.255	− 0.204	0.128	0.307	− 0.354	0.002*	0.026*	− 0.336	0.008*	0.049*

	PI			RI								
	*r*	P (unc.)	P (FDR)	*r*	P (unc.)	P (FDR)						
**Left CCA**												
MF	− 0.433	0.006*	0.050*	− 0.502	0.002*	0.040*						
IAF	− 0.327	0.022*	0.080	− 0.359	0.015*	0.071						
SE	− 0.253	0.091	0.233	− 0.396	0.016*	0.071						
Mot	− 0.161	0.231	0.352	− 0.102	0.318	0.352						
Cog	− 0.376	0.029*	0.211	− 0.318	0.035*	0.211						
**Right CCA**												
MF	− 0.187	0.143	0.214	0.083	0.277	0.312						
IAF	− 0.145	0.182	0.233	0.001	0.392	0.392						
SE	0.127	0.239	0.287	0.187	0.087	0.157						
Mot	− 0.015	0.480	0.480	− 0.025	0.389	0.424						
Cog	0.181	0.181	0.359	0.203	0.032*	0.128						

*CCA* common carotid arteries, *Cog*. cognitive-FIM score, *FIM* functional independent measurement, *IAF* individual alpha frequency, *MEG* magnetoencephalography, *MF* median frequency, *Mot* motor-FIM score, *P (FDR)* *p* values of bootstrapping statistics with FDR-correction, *P (unc.)* uncorrected *p* values of bootstrapping statistics, *PI* pulsatility index, *r* average correlation coefficient across bootstrap iterations, *RI* resistance index, *SE* Shannon entropy.

*A statistically significant correlation after FDR correction.

**Table 3:** Results of LMEM examining the effects of the left ultrasonographic parameters on MEG spectral parameters and FIM.

	DA				PSV				EDV				MV			
	*β*	*t*	P (unc.)	P (FDR)	*β*	*t*	P (unc.)	P (FDR)	*β*	*t*	P (unc.)	P (FDR)	*β*	*t*	P (unc.)	P (FDR)
**Left CCA**																
MF	1.133	1.757	0.094	0.375	− 0.049	− 2.001	0.059	0.355	0.023	0.248	0.806	0.887	− 0.050	− 0.840	0.411	0.616
IAF	0.054	0.208	0.837	0.887	− 0.010	− 0.939	0.359	0.587	0.002	0.078	0.938	0.938	− 0.009	− 0.447	0.660	0.887
SE	0.026	1.702	0.104	0.375	< 0.001	− 0.270	0.790	0.887	0.002	0.977	0.340	0.587	< 0.001	− 0.252	0.803	0.887
Mot	− 4.294	− 0.784	0.442	0.892	− 0.147	− 0.654	0.521	0.892	− 0.185	− 0.279	0.783	0.961	− 0.120	− 0.262	0.796	0.961
Cog	0.089	0.050	0.961	0.961	− 0.051	− 0.719	0.480	0.892	0.011	0.054	0.958	0.961	− 0.031	− 0.218	0.830	0.961
**Right CCA**																
MF	2.063	2.965	0.008*	0.036*	− 0.063	− 3.561	0.002*	0.023*	− 0.209	− 2.343	0.030*	0.089	− 0.148	− 3.449	0.003*	0.023*
IAF	0.636	2.181	0.041*	0.106	− 0.022	− 2.943	0.008*	0.036*	− 0.059	− 1.935	0.067	0.151	− 0.049	− 2.662	0.015*	0.054
SE	0.045	1.764	0.093	0.186	− 0.001	− 1.104	0.283	0.391	− 0.005	− 1.236	0.231	0.346	− 0.003	− 1.422	0.170	0.307
Mot	3.311	0.491	0.629	0.876	− 0.110	− 0.599	0.556	0.876	− 0.332	− 0.451	0.657	0.876	− 0.282	− 0.614	0.546	0.876
Cog	2.718	1.286	0.213	0.682	− 0.072	− 1.246	0.227	0.682	− 0.377	− 1.792	0.088	0.530	− 0.263	− 1.973	0.062	0.530

	PI				RI											
	*β*	*t*	P (unc.)	P (FDR)	*β*	*t*	P (unc.)	P (FDR)								
**Left CCA**																
MF	− 2.939	− 2.078	0.051	0.355	− 20.887	− 2.675	0.015*	0.262								
IAF	− 0.717	− 1.259	0.222	0.500	− 5.024	− 1.543	0.139	0.416								
SE	− 0.073	− 1.105	0.282	0.564	− 0.493	− 1.353	0.191	0.491								
Mot	− 15.064	− 1.250	0.226	0.892	− 59.157	− 0.829	0.417	0.892								
Cog	− 4.370	− 1.116	0.278	0.892	− 21.971	− 0.959	0.349	0.892								
**Right CCA**																
MF	− 1.753	− 0.954	0.352	0.452	0.588	0.251	0.804	0.852								
IAF	− 0.443	− 0.615	0.545	0.654	− 0.149	− 0.179	0.859	0.859								
SE	0.013	0.313	0.758	0.852	0.066	1.353	0.191	0.313								
Mot	− 1.235	− 0.088	0.931	0.931	1.899	0.108	0.915	0.931								
Cog	4.760	1.080	0.293	0.703	0.660	0.116	0.909	0.931								

*CCA* common carotid arteries, *Cog*. cognitive-FIM score, *FIM* functional independent measurement, *IAF* individual alpha frequency, *LMEM* linear mixed-effect model, *MEG* magnetoencephalography, *MF* median frequency, *Mot*. motor-FIM score, *P (FDR)* *p* values of bootstrapping statistics with FDR-correction, *P (unc.)* uncorrected *p* values of *t* test, *SE* Shannon entropy.

*β*, estimated coefficient of the predictor.

*t*, *t*-values for testing the null hypothesis that the coefficients are equal to zero.

*The terms that make significant contributions to the model.

In terms of the right CCA, ultrasonography local factors (DA, PSV, EDV, and MV) were more important (Table 2; Figs. 1b, 2a,b). DA in the right CCA positively correlated with IAF. The other three haemodynamic parameters (PSV, EDV, and MV) negatively correlated with MF and IAF, but not with SE. Neither of the downstream factors (PI and RI) correlated with any MEG spectral parameter.

**Figure 3:**
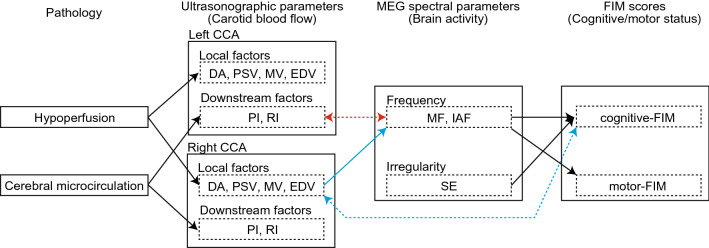
Relationships between ultrasonographic parameters, MEG spectral parameters, and FIM scores. A broken box represents subcategory of each modality. Broken arrows indicate the parameters’ significant correlations, whereas solid arrows indicate significant influences after controlling for biases in terms of patient age and sex. Red and blue arrows indicate significant associations regarding the left and right CCA, respectively (i.e*.,* asymmetrical association). Black arrows indicate significant associations not relevant to the asymmetricity of the CCA (left or right). CCA common carotid arteries, DA diameter of artery, EDV end-diastolic velocity, FIM Functional Independence Measure, IAF individual alpha frequency, MEG magnetoencepharography, MF median frequency, MV mean velocity, P I pulsatility index, PSV peak systolic flow velocity, RI resistance index, SE Shannon entropy.

EDV and MV in the right CCA significantly correlated with cognitive-FIM (Fig. 3), and the RI showed a mild correlation with cognitive-FIM (Table 2). No significant correlations were found between the other ultrasonographic parameters (DA, PSV, and the PI) in the right CCA and cognitive-FIM. When the biases from patients’ profiles had been eliminated, similar results were obtained. DA, PSV, and MV in the right CCA had a significant influence on MF, and PSV had a significant influence on IAF. EDV mildly influenced MF, and DA /MV mildly influenced IAF (P (unc.) < 0.05). No downstream factors significantly influenced MEG spectral parameters (Table 3). The relationships between right CCA parameters and cognitive-FIM did not persist after controlling for profile biases (Table 3). No significant correlation was found between ultrasonographic parameters in the right CCA and motor-FIM after controlling for profile biases (Tables 2, 3).

### Between modality association: MEG and FIM

To confirm the correlation between resting-state brain activity and cognitive status, we compared the association between MEG spectral parameters and FIM scores. Both cognitive-FIM and motor-FIM correlated with MF (cognitive-FIM: *r* = 0.509, P (FDR) = 0.027; motor-FIM: *r* = 0.440, P (FDR) = 0.027) and IAF (cognitive-FIM: *r* = 0.568, P (FDR) = 0.004; motor-FIM: *r* = 0.419, P (FDR) = 0.027). We found a correlation between SE and cognitive-FIM (*r* = 0.451, P (FDR) = 0.045), but not between SE and motor-FIM (*r* = 0.248, P (FDR) = 0.106). The same relationships were found after controlling for the patients’ profiles: both MF and IAF significantly influenced cognitive-FIM (MF: *β* = 1.299, P (FDR) = 0.020; IAF: *β* = 4.043, P (FDR) = 0.020) and motor-FIM (MF: *β* = 4.027, P (FDR) = 0.020; IAF: *β* = 10.172, P (FDR) = 0.020) (solid arrows in Fig. 3). SE had a significant influence on cognitive-FIM (*β* = 50.216, P (FDR) = 0.020) but not on motor-FIM (*β* = 88.759, P (FDR) = 0.182).

## Discussion

There are three major findings in this study. First, ultrasonographic parameters were associated with MEG spectral parameters, and MEG spectral parameters were associated with cognitive-FIM scores. However, ultrasonographic parameters were not directly associated with cognitive-FIM scores. Second, local factors in the right CCA were associated with MEG spectral parameters, whereas downstream factors in the left CCA were associated with MEG spectral parameters. Third, the influences of local factors in the right CCA remained significant even after controlling for patient age and sex. These findings supported our hypothesis, in that there was an asymmetrical association found in relation to the left and right sides of the CCA in terms of brain activity and cognitive status.

We initially discuss our results prior to controlling for biases in terms of age and sex. Within-modality correlation analysis confirmed that six ultrasonographic parameters fell into two categories^23^, namely, local factors (DA, PSV, MV, and EDV) and downstream factors (the PI and the RI). All local factors correlated with each other on both sides of the CCA (Table 1; Fig. 1). The two downstream factors correlated in the left CCA, although their relationship was not significant in the right CCA. There were several cross-factor interactions. Both downstream factors correlated with EDV and MV in the left CCA, whereas only the RI correlated with EDV in the right CCA. The asymmetry found in the relationship between ultrasonographic parameters could be due to anatomical differences: the left CCA is a branch of the aortic arch, whereas the right CCA is a branch of the brachiocephalic artery. These results indicate that the two ultrasonographic parameter categories should be considered separately. The local factors represent hypoperfusion and were associated with both MEG spectral parameters (MF and IAF) and cognitive-FIM scores in the right CCA (Table 2; Figs. 1b, 2a,b, 3). However, neither of the two downstream factors in the right CCA correlated with any MEG spectral parameters nor with cognitive-FIM. These results suggest that pathological CCA function in the right CCA impairs brain activity and cognitive status via hypoperfusion, as we anticipated. The downstream factors reflect the state of the cerebral microcirculation. They significantly correlated with atherosclerosis risk scores and the presence of brain infarction^26,27^. The PI reflects the transmission of pulsatile energy into the cerebral microcirculation. It has been shown to be positively associated with the development of stroke^28^, which is a major risk factor of dementia. A higher PI indicates a higher risk of brain microbleeds and lacunar infarcts^28,29^. The RI reflects blood flow from the microvascular bed and its values have been found to correlate with arteriosclerosis risk factors and manifestations^30,31^. The PI and the RI negatively correlated with MF in the left CCA (Table 2; Figs. 1a, 2d, 3). No local factor in the left CCA significantly correlated with any MEG spectral parameters. These results suggest that pathological cerebral microcirculation, such as microbleeds, lacunar infarcts, and arteriosclerosis (represented by downstream factors in ultrasonographic data), impaired brain activity and cognitive status to a greater extent than hypoperfusion in the left CCA. Neither downstream nor local factors were significantly associated with cognitive-FIM scores in the left CCA. Although there were few significant associations between cognitive-FIM and ultrasonographic parameters, cognitive-FIM was significantly associated with MEG spectral parameters (Fig. 3). This suggests that cognitive status (i.e*.* cognitive-FIM) was influenced by carotid blood flow (i.e*.* ultrasonographic parameters) indirectly, which was mediated by brain activity (i.e*.* MEG spectral parameters).

Age and sex are possible confounding factors because they affect cardiac functions^32^, brain activities^33^, and cognitive status^34^. After considering these potential biases, our results changed slightly. The relationship between local factors (EDV and MV) in the right CCA and cognitive-FIM was not significant (Table 3, broken blue arrow in Fig. 3), whereas the relationship had been significant prior to controlling for these profile factors (Table 2). This suggests that the relationship could be solely explained in terms of age and/or sex. Similarly, the relationships between the downstream factors in the left CCA and the MEG spectral parameter (MF) did not remain statistically significant after controlling for the profile factors (Table 3, broken red arrow in Fig. 3). There are two possible explanations for this finding. As with the relationship between local factors and cognitive-FIM in the right CCA, it could indicate that the relationship was explicable in terms of age and sex. The downstream factors correlated with arteriosclerosis risk factors, which include age^35^. Alternatively, it might have been due to our statistical strategy. Examining the null-hypothesis in which the coefficient of the RI (predictor) for predicting MF was equal to zero, the *t*-value yielded a statistically significant level in a single test (P (unc.) = 0.015). It is plausible to consider that this significant result was masked due to the FDR correction. Since this study is examining a new field, we acknowledge that the procedure we adopted is exploratory, and that certain significant associations may not have been detected. However, the influence of local factors in the right CCA on MEG spectral parameters remained significant even with strict correction, which at least supported our hypothesis, namely, that the left and right side of the CCA had an asymmetrical association with brain activity.

The observed asymmetry may have important implications for clinical practice. Blood flow in the CCA can be improved through surgical treatment such as carotid endarterectomy or carotid artery stenting. Both techniques have been reported to improve blood flow and cognitive function, although this remains controversial^36–39^. However, our study findings indicated that improvement in the blood flow (i.e*.* affecting local factors) of CCA may not improve cognitive function, because blood flow was not significantly associated with brain activity in the left CCA. Therefore, consideration should be given to differences in the relationship between the left and right CCA and brain activity and cognitive function when designing a therapeutic strategy for pathological CCA.

In our study, we report two further findings concerning the relationships between CCA blood flow, resting-state brain activity, and cognitive/motor status. First, the relationship between carotid blood flow and cognitive status was found to be indirect and mediated through brain activity. We presumed that ultrasonographic parameters would be associated with cognitive-FIM because cerebral hypoperfusion affects cognitive status, measured using MMSE^10^, which has been found to correlate with cognitive-FIM scores^40^. However, our results did not support this presumption. The significant relationships between ultrasonographic parameters and cognitive-FIM scores were not replicated after controlling for profile biases. It is plausible to consider that the previously reported relationship between carotid blood flow (i.e*.* ultrasonographic parameters) and cognitive status (i.e*.* cognitive-FIM) was mediated through brain activity (i.e*.* MEG spectral parameters). This result suggests that MEG spectral parameters comprise more risk factors, which are represented as changes in blood flow in the CCA, than the FIM score, given that both cognitive-FIM and motor-FIM correlated significantly with MF and IAF, whereas correlations between cognitive-FIM and motor-FIM and between motor-FIM and ultrasonographic parameters were not significant. This implies that cognitive-FIM and motor-FIM are more closely linked to resting-state brain activities (i.e*.* MEG spectral parameters) than carotid blood flow (i.e*.* ultrasonographic parameters). Concerning the lack of association found between ultrasonographic parameters and cognitive-FIM scores, the ceiling effect and cognitive reserve provide two possible explanations. The cognitive-FIM scores were influenced by the ceiling effect, as evident in Fig. 2c,f^41^. This indicates that cognitive-FIM failed to capture continuous gradients in relation to cognitive status, especially for those patients whose cognitive functions were largely retained; therefore, the lack of association between ultrasonographic parameters and cognitive-FIM scores occurred because the scores did not reflect cognitive status appropriately. However, MEG spectral parameters were not similarly limited, which may explain why they were more sensitive to detecting mild cognitive impairment than FIM. Another explanation involves cognitive reserve, which has often been used to explain variations in severity between pathological brain damage and cognitive impairment^42^. Individual differences in cognitive reserve may explain some of the variation found in this study. Second, SE was less sensitive to the change in resting-state brain activity due to impaired carotid blood flow (Figs. 1, 3). No significant association was found between SE and ultrasonographic parameters (Table 2, 3). All three MEG spectral parameters (MF, IAF, and SE) were calculated from the same normalised power spectral density (PSDn) of the MEG signals, and within-modality association analysis indicated that they correlated significantly, except between IAF and SE. However, these parameters can behave differently because they represent different properties of the PSDn. MF is an index that summarises the power balance between slow and fast neural oscillations, including alpha oscillations. IAF is another index that represents the peak frequency of alpha oscillations. MF and IAF are slightly overlapping, and we consider it reasonable to suggest that they positively correlated with each other. In contrast, SE provides complementary information to that yielded using MF and IAF, although SE is calculated from the same PSDn. SE quantifies the irregularity of the PSDn and reflects the diversity of neural oscillatory components. Although previous research has shown that all three MEG spectral parameters are sensitive to changes in resting-state brain activity due to cognitive impairment^17,43–48^, most previous studies were conducted involving patients with Alzheimer's disease, not in those with vascular dementia. A previous study using electroencephalography showed that the properties of the power spectral density differ between Alzheimer’s disease and vascular dementia^49^. Hence, we speculated that SE was less sensitive to changes in resting-state brain activity due to vascular dementia.

This study had some limitations. First, data were acquired from patients who had been admitted to our hospital due to symptomatic cerebrovascular diseases. Patients with right hemisphere stroke tend to show fewer symptoms because the right hemisphere is considered the ‘minor hemisphere’ in most cases^13^. Moreover, such patients may have had more severe atherosclerosis and stroke at admission, which could have led to the asymmetry observed in our study. Furthermore, no healthy volunteers (i.e*.* a control group) were enrolled in our study because we focused on the pathological effects of cerebral blood flow rather than the physiological effects. To address these limitations, we have begun another study involving volunteers with no symptomatic stroke. Second, we did not consider arteriosclerosis in the cerebral arteries or other arteries, such as the vertebral arteries. Moreover, we did not consider the side and location of the lesions in the brain because they were heterogenous and often temporally and spatially multiple. Clinical magnetic resonance angiography results occasionally showed severe stenosis in cerebral arteries contralateral to the lesion-affected side. Occasionally, lesions were also located outside of the cerebrum (Supplementary Table 1) and, consequently, determining the affected side of the hemisphere was challenging. However, despite this limitation, asymmetry was apparent. Third, cognitive status was only assessed using cognitive-FIM, whereas other neuropsychological assessments such as the MMSE were not used (see the Methods section). In this retrospective study of clinical data, we did not undertake a cognitive assessment; however, our main findings indicating a correlation between ultrasonographic parameters and MEG spectral parameters and a correlation between cognitive-FIM scores and asymmetry between the left and right CCA were not affected. Fourth, our sample size was limited. Given the retrospective design of this clinical study, identifying patients who had undergone both ultrasonography and MEG was challenging as few patients with cerebrovascular diseases had undergone both examinations due to clinical limitations, such as the capacity of the clinical laboratory, or limitations in the health insurance system. To address this fourth limitation, we used a nonparametric bootstrapping approach for the correlation analysis. Given the high effect sizes (*r* in the correlation analysis was > 0.3 in terms of absolute value, in the most significant cases) and stable results across different statistical techniques (i.e*.* bootstrapping correlation and LMEM approaches: see “Methods” section for details), the results can be considered reliable. We consider that our study findings may help clinicians understand the importance of ultrasonography and MEG, which may encourage data collection and increase sample sizes in future studies. Finally, carotid ultrasonography measurements, MEG recordings, and FIM assessments had all been undertaken on different days. This was also due to limitations concerning data collection during daily clinical practice. In future studies, a controlled data collection procedure is needed to support our findings.

In conclusion, our study findings showed that pathological CCA on the left and right sides of the brain were differently associated with resting-state brain activity and cognitive status. Resting-state brain activity and cognitive status were associated with downstream factors (i.e*.* the PI and the RI) in the left CCA whereas, in the right CCA, they were associated with local factors (i.e*.* DA, PSV, MV, and EDV). This result indicates that hypoperfusion in the right CCA and cerebral microcirculation in the left CCA influenced brain activity and cognitive status. Consequently, therapeutic strategies to prevent cognitive impairment need to be different when targeting the left and right CCA.

## Methods

### Participants

Carotid ultrasonography and MEG data, and the FIM scores of 23 participants (women, n = 6; mean age ± standard deviation, 67.6 ± 13.6 years [range 35–88 years]) at the Kumagaya General Hospital (Japan) were retrospectively analysed. The patients were admitted to the Department of Neurosurgery at our hospital due to cerebrovascular diseases (ischemic stroke, n = 19; transient ischaemic attack, n = 4). The clinical profile details are shown in Supplementary Table 1. Our investigation was conducted according to the Declaration of Helsinki, in accordance with national and international guidelines. The study was approved by the Ethics Committee of Kumagaya General Hospital (approval number: 47). The committee granted the use of clinical data for this retrospective study unless patients did not agree to our re-use of their data. Written informed consent was obtained if a patient’s cognitive/physical condition allowed.

### Carotid ultrasonography

Carotid blood flow was evaluated using carotid ultrasonography as part of the clinical practice. The ultrasonography system used was ARIETTA 70 or Noblus (Hitachi, Tokyo, Japan). Six ultrasonographic parameters (DA, PSV, EDV, MV, PI, and RI) of the CCA were measured on both sides (left and right CCA). Following findings from a previous study that showed an association between blood flow velocity in the CCA (but not in the internal carotid artery) and cognitive performance^10^, we chose to evaluate the CCA instead of the internal carotid artery, which is downstream of the CCA and makes contact with the cerebral arteries directly. In each patient, six raw ultrasonographic parameters (DA, PSV, EDV, MV, PI, and RI) for each side of the CCA (left and right) were used for statistical analysis.

### MEG scanning

The scanning and analysis procedures of MEG activity followed the procedure applied in our previous study^15^. Spontaneous neural oscillations were recorded for 5 min to screen for epilepsy and evaluate brain state as part of the clinical practice. The MEG system used was a 160-channel whole-head type (RICOH160-1; RICOH, Tokyo, Japan), placed in a magnetically shielded room. During the scan, the patients were asked to remain relaxed in a supine position with their eyes closed. The sensor coils were gradiometers with a diameter of 15 mm and a height of 50 mm. Each pair of sensor coils was separated at a 23-mm distance. The sampling frequency was 2,000 Hz with a 500-Hz low-pass filtering during the recording.

### MEG data analysis

MEG data were pre-processed offline using the software package SPM-12 (Wellcome Trust Centre for Neuroimaging, London, UK; https://www.fil.ion.ucl.ac.uk/spm/). Given that MEG spectral parameters are sensitive to artefacts, they were manually removed through applying a principal component analysis, if necessary. For this task, analysis software provided by the MEG manufacturer was used. Power line noise was removed using a 50-Hz band-stop filter. For ease of analysis, the continuous MEG signals were divided into 10-s segments. The segments in which the magnetic signal exceeded 6,000 fT were discarded, when applicable. Thereafter, different properties of spontaneous neural oscillations were quantified using the following three MEG spectral parameters: MF, IAF, and SE^17,24,25^. These MEG-based parameters were computed from the power spectral density (PSD). The PSD was estimated using the Blackman-Tukey method through utilising non-overlapping 10-s segments. To obtain the normalised PSD (PSDn), the original PSD was divided by the power in the frequency range of interest, i.e*.* 1–70-Hz^50^.

The first parameter, namely, MF, quantifies the frequency lying at the midpoint of the PSDn (i.e*.* the median of the frequency distribution represented by the PSDn). MF is a useful spectral parameter to summarise the slowing of spontaneous neural oscillatory activity in patients with dementia^17^ as it reflects the typical increase of low frequency oscillatory components, along with the decrease of high frequency neural activity in patients with cognitive impairment. The second parameter, namely, IAF, provides supplementary information to MF. It is computed as the frequency at which the peak of the PSDn in the alpha band is observed^17^. IAF is the parameter that characterises dominant alpha activity, typically observed in human adults in the eyes-closed resting condition, although it gradually alters during dementia progression^17^. The third parameter, namely, SE, yields a complementary description of the PSDn to MF and IAF. Specifically, SE quantifies the irregularity of the frequency distribution of the oscillatory components represented in the PSDn^17,25^. Hence, it provides an estimation of the irregularity of MEG activity. Interestingly, previous studies have showed that SE can reflect the loss of irregularity associated with the progression of dementia^17,25^.

These spectral parameters were calculated for each 10-s epoch and MEG sensor, after which they were averaged across epochs and sensors to generate three representative MEG spectral parameters for each patient.

### Cognitive and motor status assessment

Patients' cognitive and motor statuses were assessed using the FIM during their admission as a part of the clinical practice. The FIM is an assessment tool for cognitive and motor status during rehabilitation for stroke^22^. The FIM is the most commonly used test in stroke units and rehabilitation departments in Japan to evaluate and monitor a patient’s condition, and its use is strongly recommended (and is virtually mandatory) by the Japanese Ministry of Health, Labour and Welfare. The FIM is advantageous when it is used for admitted patients with cerebrovascular diseases because it does not require any patient effort. It can even be used for patients with severe cognitive impairment, paralysis, or aphasia. Most other neuropsychological assessments cannot properly be used for these patients. The reliability and validity of the FIM have previously been well established in the field of rehabilitation^51,52^. The FIM consists of two domains: cognitive-FIM and motor-FIM. The maximum possible cognitive-FIM and motor-FIM scores are 35 points and 83 points, respectively, with a lower score indicating more severe impairment. The date difference between a FIM assessment and a MEG scan was 31.7 ± 27.9 days (range 0–108 days).

### Statistical analysis

To examine the relationship between data modalities (i.e*.* ultrasonographic parameters, MEG spectral parameters, and FIM scores), a correlation analysis was performed using a non-parametric bootstrapping approach. Bootstrapping statistics have methodological advantages over classical statistical inference (e.g. the Gaussian assumption)^53^. The correlations were examined *within* modalities and *between* modalities, separately. For each pair of variables, Pearson’s coefficient was calculated through resampling with replacement data across all patients 20,000 times. The percentage of the resampled coefficients, when larger or smaller than 0 (the smaller value), was taken as the significance level (P value). We report the grand mean of the correlation coefficient (*r*) across bootstrap iterations and P values.

As an extension of the correlation analysis, possible biases in relation to patient age and sex were considered. Bias-free *between-*modality relationships were examined using an LMEM analysis. An LMEM analysis assesses directional relationships; therefore, we made the following assumptions according to physiological rationales: (1) ultrasonographic parameters (predictor variables) influence MEG spectral parameters and FIM scores (response variables), and (2) MEG spectral parameters (predictor variables) influence FIM scores (response variables). For each pair of variables, the response variable was subjected to LMEM with two fixed covariates, one of which was a predictor variable and the other was patient age. To consider sex differences, a random intercept and random slopes (for both fixed predictors) were entered into the model for each sex. The model was estimated using a maximum likelihood method. Estimated fixed coefficients (*β*) of fixed predictors were tested for a null hypothesis that the coefficients would be equal to zero, using a *t* test.

Since the analyses were exploratory and generated matrices (Tables 1, 2, 3) where each of the statistical values were tested against our null hypothesis (that the coefficients would be equal to zero), this series of results was at risk of an increasing Type-I error^54^. To manage this risk, we reported P values controlled for the false detection rate (FDR) using the Benjamini–Hochberg method^55^. Although we considered P (FDR-corrected) < 0.05 as statistically significant, P (uncorrected) < 0.05 has also been reported to indicate mild correlations/influences between tested parameters. Significant values are marked with asterisks in Tables 1, 2 and 3. All statistical analyses were performed using the Statistics and Machine Learning Toolbox and Multiple Testing Toolbox^56^ in MATLAB (MathWorks, Natick, MA) software.

### Ethics approval and consent to participate

Reuse of the data for the present study was approved by the ethics committee of Kumagaya General Hospital (#47). Additionally, written informed consents for using data were obtained as far as patients’ cognitive/physical condition allowed.

## Supplementary Information

Supplementary Information 1.

## Data Availability

The dataset analysed during the current study is available in the repository, Shigihara, Yoshihito, 2021, "Replication Data for: Carotid ultrasonography and resting-state magnetoencephalography", 10.7910/DVN/DQOLFS, Harvard Dataverse.
