# Publisher Correction: The association between carotid blood flow and resting-state brain activity in patients with cerebrovascular diseases

**DOI:** 10.1038/s41598-021-97494-y

**Published:** 2021-09-01

**Authors:** Takahiro Matsumoto, Hideyuki Hoshi, Yoko Hirata, Sayuri Ichikawa, Keisuke Fukasawa, Tomoyuki Gonda, Jesús Poza, Víctor Rodríguez-González, Carlos Gómez, Yoshihito Shigihara

**Affiliations:** 1 Department of Neurosurgery, Kumagaya General Hospital, Kumagaya, 360-8567 Japan; 2 Precision Medicine Centre, Hokuto Hospital, Kisen-7-5 Inadacho, Obihiro, Hokkaido 080-0833 Japan; 3 Clinical Laboratory, Kumagaya General Hospital, Kumagaya, 360-8567 Japan; 4 Department of Rehabilitation, Kumagaya General Hospital, Kumagaya, 360-8567 Japan; 5 Biomedical Engineering Group, Higher Technical School of Telecommunications Engineering, University of Valladolid, Castilla y León, 47011 Valladolid, Spain; 6 Centro de Investigación Biomédica en Red en Bioingeniería, (CIBER-BBN), Biomateriales y Nanomedicina, Castilla y León, 47011 Valladolid, Spain; 7 Instituto de Investigación en Matemáticas (IMUVA), University of Valladolid, Castilla y León, 47011 Valladolid, Spain; 8 Precision Medicine Centre, Kumagaya General Hospital, Kumagaya, 360-8567 Japan

Correction to: *Scientific Reports* https://doi.org/10.1038/s41598-021-94717-0, published online 27 July 2021

The original version of this Article contained errors in the arrangement of Tables 1, 2 and 3 where the heading rows “Left CCA” and “Right CCA” were incorrectly split as “Left”, “CCA” and “Right”, “CCA”.

The original Tables 1, 2 and 3 and accompanying legends appear below.

**Table 1:** Correlation matrix within sided ultrasonographic parameters.

	DA			PSV			EDV			MV			PI		
	*r*	P (unc.)	P (FDR)	*r*	P (unc.)	P (FDR)	*r*	P (unc.)	P (FDR)	*r*	P (unc.)	P (FDR)	*r*	P (unc.)	P (FDR)
**Left**															
PSV	− 0.644	< 0.001*	< 0.001*												
**CCA**															
EDV	− 0.339	0.035*	0.047*	0.646	< 0.001*	< 0.001*									
MV	− 0.548	0.001*	0.002*	0.793	< 0.001*	< 0.001*	0.907	< 0.001*	< 0.001*						
PI	− 0.054	0.388	0.416	0.083	0.346	0.400	− 0.632	< 0.001*	< 0.001*	− 0.492	0.003*	0.005*			
RI	− 0.132	0.271	0.339	0.027	0.446	0.446	− 0.712	< 0.001*	< 0.001*	− 0.475	0.007*	0.011*	0.927	< 0.001*	< 0.001*
**Right**															
PSV	− 0.634	< 0.001*	< 0.001*												
**CCA**															
EDV	− 0.660	< 0.001*	0.001*	0.809	< 0.001*	< 0.001*									
MV	− 0.722	< 0.001*	< 0.001*	0.923	< 0.001*	< 0.001*	0.944	< 0.001*	< 0.001*						
PI	0.252	0.104	0.196	0.152	0.235	0.320	− 0.175	0.161	0.243	− 0.107	0.256	0.320			
RI	− 0.055	0.335	0.377	0.110	0.162	0.243	− 0.310	0.002*	0.003*	− 0.083	0.394	0.394	− 0.018	0.352	0.377

*DA* diameter of artery, *CCA* common carotid arteries, *EDV* end-diastolic velocity, *MV* mean velocity, *P (unc.)* uncorrected *p* values of bootstrapping statistics, *PI* pulsatility index, *r* average correlation coefficient across bootstrap iterations, *PSV* peak systolic flow velocity, *RI* resistance index, *P (FDR)* *p* values of bootstrapping statistics with FDR-correction.

*A statistically significant correlation after FDR correction.

**Table 2:** Correlation matrix between sided ultrasonographic parameters, MEG spectral parameters, and FIM.

	DA			PSV			EDV			MV		
	*r*	P (unc.)	P (FDR)	*r*	P (unc.)	P (FDR)	*r*	P (unc.)	P (FDR)	*r*	P (unc.)	P (FDR)
**Left**												
MF	0.262	0.154	0.284	− 0.231	0.158	0.284	0.169	0.187	0.305	− 0.014	0.467	0.480
**CCA**												
IAF	− 0.003	0.480	0.480	− 0.143	0.273	0.351	0.125	0.260	0.351	0.028	0.432	0.480
SE	0.293	0.107	0.241	0.063	0.368	0.442	0.288	0.045*	0.135	0.126	0.254	0.351
Mot	− 0.130	0.255	0.352	− 0.161	0.215	0.352	− 0.109	0.313	0.352	− 0.114	0.303	0.352
Cog	− 0.070	0.323	0.352	− 0.025	0.461	0.461	0.217	0.130	0.352	0.179	0.162	0.352
**Right**												
MF	0.419	0.042*	0.095	− 0.484	0.007*	0.024*	− 0.421	0.013*	0.039*	− 0.506	0.004*	0.024*
**CCA**												
IAF	0.358	0.018*	0.046*	− 0.480	0.005*	0.024*	− 0.390	0.006*	0.024*	− 0.475	0.002*	0.024*
SE	0.332	0.055	0.110	− 0.094	0.325	0.344	− 0.205	0.178	0.233	− 0.248	0.114	0.186
Mot	0.101	0.299	0.359	− 0.125	0.251	0.359	− 0.110	0.281	0.359	− 0.142	0.230	0.359
Cog	0.177	0.085	0.255	− 0.204	0.128	0.307	− 0.354	0.002*	0.026*	− 0.336	0.008*	0.049*

	PI			RI								
	*r*	P (unc.)	P (FDR)	*r*	P (unc.)	P (FDR)						
**Left**												
MF	− 0.433	0.006*	0.050*	− 0.502	0.002*	0.040*						
**CCA**												
IAF	− 0.327	0.022*	0.080	− 0.359	0.015*	0.071						
SE	− 0.253	0.091	0.233	− 0.396	0.016*	0.071						
Mot	− 0.161	0.231	0.352	− 0.102	0.318	0.352						
Cog	− 0.376	0.029*	0.211	− 0.318	0.035*	0.211						
**Right**												
MF	− 0.187	0.143	0.214	0.083	0.277	0.312						
**CCA**												
IAF	− 0.145	0.182	0.233	0.001	0.392	0.392						
SE	0.127	0.239	0.287	0.187	0.087	0.157						
Mot	− 0.015	0.480	0.480	− 0.025	0.389	0.424						
Cog	0.181	0.181	0.359	0.203	0.032*	0.128						

*CCA* common carotid arteries, *Cog*. cognitive-FIM score, *FIM* functional independent measurement, *IAF* individual alpha frequency, *MEG* magnetoencephalography, *MF* median frequency, *Mot* motor-FIM score, *P (FDR)* *p* values of bootstrapping statistics with FDR-correction, *P (unc.)* uncorrected *p* values of bootstrapping statistics, *PI* pulsatility index, *r* average correlation coefficient across bootstrap iterations, *RI* resistance index, *SE* Shannon entropy.

*A statistically significant correlation after FDR correction.

**Table 3:** Results of LMEM examining the effects of the left ultrasonographic parameters on MEG spectral parameters and FIM.

	DA				PSV				EDV				MV			
	*β*	*t*	P (unc.)	P (FDR)	*β*	*t*	P (unc.)	P (FDR)	*β*	*t*	P (unc.)	P (FDR)	*β*	*t*	P (unc.)	P (FDR)
**Left**																
MF	1.133	1.757	0.094	0.375	− 0.049	− 2.001	0.059	0.355	0.023	0.248	0.806	0.887	− 0.050	− 0.840	0.411	0.616
**CCA**																
IAF	0.054	0.208	0.837	0.887	− 0.010	− 0.939	0.359	0.587	0.002	0.078	0.938	0.938	− 0.009	− 0.447	0.660	0.887
SE	0.026	1.702	0.104	0.375	< 0.001	− 0.270	0.790	0.887	0.002	0.977	0.340	0.587	< 0.001	− 0.252	0.803	0.887
Mot	− 4.294	− 0.784	0.442	0.892	− 0.147	− 0.654	0.521	0.892	− 0.185	− 0.279	0.783	0.961	− 0.120	− 0.262	0.796	0.961
Cog	0.089	0.050	0.961	0.961	− 0.051	− 0.719	0.480	0.892	0.011	0.054	0.958	0.961	− 0.031	− 0.218	0.830	0.961
**Right**																
MF	2.063	2.965	0.008*	0.036*	− 0.063	− 3.561	0.002*	0.023*	− 0.209	− 2.343	0.030*	0.089	− 0.148	− 3.449	0.003*	0.023*
**CCA**																
IAF	0.636	2.181	0.041*	0.106	− 0.022	− 2.943	0.008*	0.036*	− 0.059	− 1.935	0.067	0.151	− 0.049	− 2.662	0.015*	0.054
SE	0.045	1.764	0.093	0.186	− 0.001	− 1.104	0.283	0.391	− 0.005	− 1.236	0.231	0.346	− 0.003	− 1.422	0.170	0.307
Mot	3.311	0.491	0.629	0.876	− 0.110	− 0.599	0.556	0.876	− 0.332	− 0.451	0.657	0.876	− 0.282	− 0.614	0.546	0.876
Cog	2.718	1.286	0.213	0.682	− 0.072	− 1.246	0.227	0.682	− 0.377	− 1.792	0.088	0.530	− 0.263	− 1.973	0.062	0.530

	PI				RI											
	*β*	*t*	P (unc.)	P (FDR)	*β*	*t*	P (unc.)	P (FDR)								
**Left**																
MF	− 2.939	− 2.078	0.051	0.355	− 20.887	− 2.675	0.015*	0.262								
**CCA**																
IAF	− 0.717	− 1.259	0.222	0.500	− 5.024	− 1.543	0.139	0.416								
SE	− 0.073	− 1.105	0.282	0.564	− 0.493	− 1.353	0.191	0.491								
Mot	− 15.064	− 1.250	0.226	0.892	− 59.157	− 0.829	0.417	0.892								
Cog	− 4.370	− 1.116	0.278	0.892	− 21.971	− 0.959	0.349	0.892								
**Right**																
MF	− 1.753	− 0.954	0.352	0.452	0.588	0.251	0.804	0.852								
**CCA**																
IAF	− 0.443	− 0.615	0.545	0.654	− 0.149	− 0.179	0.859	0.859								
SE	0.013	0.313	0.758	0.852	0.066	1.353	0.191	0.313								
Mot	− 1.235	− 0.088	0.931	0.931	1.899	0.108	0.915	0.931								
Cog	4.760	1.080	0.293	0.703	0.660	0.116	0.909	0.931								

*CCA* common carotid arteries, *Cog*. cognitive-FIM score, *FIM* functional independent measurement, *IAF* individual alpha frequency, *LMEM* linear mixed-effect model, *MEG* magnetoencephalography, *MF* median frequency, *Mot*. motor-FIM score, *P (FDR)* *p* values of bootstrapping statistics with FDR-correction, *P (unc.)* uncorrected *p* values of *t* test, *SE* Shannon entropy.

*β*, estimated coefficient of the predictor.

*t*, *t*-values for testing the null hypothesis that the coefficients are equal to zero.

*The terms that make significant contributions to the model.

The original Article has now been corrected.

