# Supplementary material for: The association between carotid blood flow and resting-state brain activity in patients with cerebrovascular diseases
Source: Sci Rep. 2021 Jul 27;11:15225. doi: 10.1038/s41598-021-94717-0 (PMC8316461; doi:10.1038/s41598-021-94717-0)
Supplement: Supplementary file 1 — Supplementary Information 1. [file 41598_2021_94717_MOESM1_ESM.docx]

**Supplementary Information**

**The association between carotid blood flow and resting-state brain activity**

**in patients with cerebrovascular disease**

Takahiro Matsumoto1, Hideyuki Hoshi2, Yoko Hirata1, Sayuri Ichikawa3, Keisuke Fukasawa3, Tomoyuki Gonda4, Jesús Poza5,6,7, Víctor Rodríguez-González5,6, Carlos Gómez5,6 ,

Yoshihito Shigihara2,8*

^1^Department of Neurosurgery, Kumagaya General Hospital, Japan

^2^Precision Medicine Centre, Hokuto Hospital, Japan

^3^Clinical Laboratory, Kumagaya General Hospital, Japan

^4^Department of Rehabilitation, Kumagaya General Hospital, Japan

^5^Biomedical Engineering Group, University of Valladolid, 47011 Valladolid, Spain

^6^Centro de Investigación Biomédica en Red en Bioingeniería, Biomateriales y Nanomedicina, (CIBER-BBN), Spain

^7^Instituto de Investigación en Matemáticas (IMUVA), University of Valladolid, 47011 Valladolid, Spain

^8^Precision Medicine Centre, Kumagaya General Hospital, Japan

Supplementary Table 1. Participant clinical profile

| ID | Age | Sex | Chief complaint | Diagnose | Responsible lesions |
| --- | --- | --- | --- | --- | --- |
| 1 | 49 | M | Left paralysis | Ischemic stroke | Right frontal lobe |
| 2 | 64 | M | Vertigo | Ischemic stroke | Left cerebellum |
| 3 | 50 | M | Aphasia and dysarthria | Ischemic stroke | Left frontal lobe |
| 4 | 78 | M | Left paralysis | Ischemic stroke | Right motor cortex |
| 5 | 82 | F | Convulsion and aphasia | Ischemic stroke | Not identified |
| 6 | 60 | M | Headache | Ischemic stroke | Not identified |
| 7 | 50 | F | Left paralysis | Ischemic stroke | Right motor cortex and parietal lobe |
| 8 | 72 | M | Left paralysis and dysarthria | Ischemic stroke | Right temporal lobe and corona radiata |
| 9 | 35 | F | Vertigo and vomiting | Ischemic stroke | Right cerebellum |
| 10 | 74 | M | Left paralysis and dysarthria | Ischemic stroke | Right medulla oblongata |
| 11 | 77 | F | Right paralysis | Ischemic stroke | Cerebellum |
| 12 | 58 | M | Left paralysis and aphasia | Ischemic stroke | Right frontal, parietal lobes and corona radiata |
| 13 | 74 | M | Left paralysis and dysarthria | Ischemic stroke | Multiple infraction in the right hemisphere |
| 14 | 79 | M | Left paralysis | TIA | Not identified |
| 15 | 71 | F | Left paralysis | TIA | Not identified |
| 16 | 72 | M | Left paralysis | Ischemic stroke | Left corona radiata and internal capsule |
| 17 | 82 | M | Aphasia and impaired consciousness | Ischemic stroke | Left frontal and occipital lobe |
| 18 | 70 | F | Dysarthria, and impaired consciousness | TIA | Not identified |
| 19 | 65 | M | Right paralysis | TIA | Not identified |
| 20 | 49 | M | Dysarthria and facial paresthesia | Ischemic stroke | Left corona radiata and putamen |
| 21 | 88 | M | Dysarthria and impaired consciousness | Ischemic stroke | Left temporal, insula, and corona radiata |
| 22 | 78 | M | Left paralysis, dysarthria, and agnosia | Ischemic stroke | Right basal ganglia and corona radiata |
| 23 | 78 | M | Disorientation and impaired consciousness | Ischemic stroke | Right frontal and corona radiata |

F, female; M, male; TIA, transient ischemic attack
